# Dielectric singularity in hyperbolic metamaterials: the inversion point of coexisting anisotropies

**DOI:** 10.1038/srep20002

**Published:** 2016-02-02

**Authors:** V. Caligiuri, R. Dhama, K. V. Sreekanth, G. Strangi, A. De Luca

**Affiliations:** 1 Department of Physics and CNR - Nanotec, University of Calabria, 87036 Rende Italy; 2 Department of Physics, Case Western Reserve University, 44106-7079 Cleveland, USA

## Abstract

Hyperbolic Metamaterials are artificially engineered materials whose optical properties can be specifically tailored to manifest an extremely high level of anisotropy. Due to this remarkable anisotropy they represent a unique opportunity to realize effective bulk meta-structure with extraordinary optical properties in the visible range. A simultaneous dielectric singularity in the in plane permittivity, with respect to the propagation direction, has to lead to a complete sign inversion of the same permittivity for that specific visible frequency. Such a drastic phase change has been theoretically highlighted in the past as the major challenge to be overcome in order to unlock many remarkable optical properties not present artificial optical systems. In this paper we experimentally demonstrate the realization of a metal-dielectric multilayer structure showing an inversion point of coexisting anisotropies at a specified wavelength in the visible range, rising from the particular design and fabrication process. Theoretical models and numerical simulations are in very good agreement with experimental data. Ellipsometrical experiments and optical modeling demonstrate the drastic type I/type II transition. Supercollimation effect has been achieved at the inversion point of the coexisting extreme anisotropies, whereas at the epsilon near zero and pole frequency the perfect lens behavior has been observed.

Metamaterials are a class of artificial materials having structural parameters much smaller than the operative wavelength, being usually nanostructured for optical frequencies. Nonetheless, their effective response to ligthwaves shows extraordinary and fascinating properties. Many promising physical behaviors can arise from materials with such unusual electromagnetic response, among which negative refraction, optical cloacking, super resolution imaging, ultra compact optical circuits, plasmonic nanolasers, inversion of the Cherenkov radiation are only few examples^1^,^2^,^3^,^4^,^5^,^6^,^7^,^8^,^9^,^10^,^11^,^12^,^13^,^14^,^15^. Recently, a new branch of the metamaterial class is receiving an increasing attention in the scientific panorama, which holds an ultra anisotropic behavior not yet found in nature for optical frequencies. These materials are known as hyperbolic metamaterials (HMMs), which are characterized by a hyperbolic dispersion determined by their effective dielectric tensor 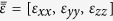. Considered as the ultra-anisotropic limit of traditional uniaxial crystals^16^, they possess one of the principal components of their permittivity tensor that is opposite in sign to the other two; in particular the in-plane isotropic components are 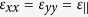, the out of plane component is 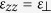 and 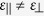. This means that the same material can interact with the radiation behaving as a metal or as a dielectric depending on the orientation of the radiation wavevector 
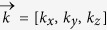
. HMMs unique properties result from the isofrequency surface of an extraordinary wave (TM polarized) propagating into the structure; in this case the dispersion relation becomes:


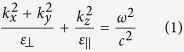


Here, 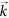 is the propagating wavevector, *ω* is the radiation frequency and *c* is the velocity of light in free space. Hyperbolic metamaterials represent one of the most promising way to practically fabricate bulk metastructures with the desired anisotropy. Specifically, two different types of geometries have been found to act as an extreme anisotropic metamaterial, showing completely opposite behaviour: metal-dielectric periodic stacks and metal nanowire arrays embedded in a dielectric matrix^17^,^18^,^19^. By properly engineering the metal/dielectric fill fraction inside the metamaterial, 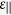 and 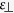 can become either positive or negative, opening the way to a wide design flexibility. The anisotropy of the device depends, in general, on the sign of the product 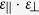, that can be 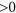 or, more interestingly, 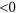. In the latter case two scenarios can occur: (i) *type I* - HMM and (ii) *type II* - HMM. In the case of *type I* - HMM 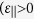 and 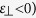, the stack behaves as a dielectric in the *xy* plane and a metal in the *z* direction, whereas the isofrequency surfaces assume the shape of an open bounded hyperboloid. However, the behavior is completely reversed and isofrequency surface became a continuous hyperboloid in the case of *type II* - HMM. As it is well known, multilayer stacks show a *type II* behavior only, and it is needed a metallic nanowire array immersed in a dielectric host to obtain a *type I* behavior. The coexistence of the two inverse anisotropies in the same metamaterial, with a zero dielectric or metal gap between them, represents an experimental challenge. Recently, Shekhar *et al*. theoretically reported a multilayer system presenting both the anisotropies, either separated by a dielectric gap or adjacent each other, showing that an experimental result with a simultaneous type I/type II behavior at the same frequency in the visible range would be not easy to achieve^20^. In the same work, an 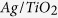 multilayer is theoretically investigated as a structure able to eliminate the dielectric gap between the type I and type II regions, enabling novel phenomena and applications. Anyway the amorphous phase of a real layer of 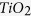 possesses optical constants drastically lower than in the birefringent phase, thus pushing the experimental transition wavelength deep in the UV (see supplementary information) and limiting the possibility of an effective implementation of such a structure in the experimental framework. A similar behavior has been observed even in the field of semiconductors. Hoffman *et al*.^21^ realized a semiconductor multilayer working at the transition wavelength of typeI/type II in the microwave range, while Silveirinha *et al*.^22^ found a comparable mechanism governing the propagation of electron waves inside a semiconductor hypergrating. In that case the coexistence of ultra-anisotropies is referred to the overall effective mass of electrons instead of the homogenized optical constants of the metastructure.

In this work we report on the design, fabrication and characterization of an epsilon near zero and pole 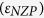 multilayer HMM, showing simultaneously 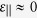 and *ε*_⊥_ ≈ ∞ within the visible range, able to overcome all of the above mentioned challenges. The realized structure, due to the careful choice of its fundamental building blocks, manifests a type I behavior covering about 90 nm range until a wavelength at which the extreme anisotropy completely reverses (414 nm), giving rise to a more common type II HMM. We also go through the experimental demonstrations of the most important behaviors of such a metamaterial. An ellipsometric analysis reveals that, at the transition wavelength between type I and type II, a dielectric singularity in the optical constants occurs and the material undergoes a dramatic phase change, which opens the way to fascinating physical phenomena. Indeed, in order to show the potential of the presented configuration, we experimentally study the supercollimation effect at the type I/type II transition wavelength by means of a confocal microscopy study. We demonstrate the possibility to harness this effect to realize an ultra-subwavelength waveguide in the metamaterial bulk, being able to keep the light beam extremely confined inside the structure for more than 100 Rayleigh lengths. We further demonstrate the near field perfect lens behavior by means of numerical simulations corroborated by an experimental proof of concept of the proposed phenomenon.

For a multilayer structure, the effective dielectric permittivities are complex quantities and, in order to evaluate their complete expressions, we refer to an extended Effective Medium Theory (EMT). This permits to include the imaginary parts of the permittivities of the main HMM building blocks:


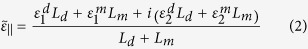



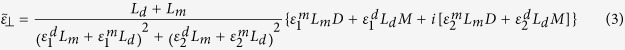


Here subscript 1 and 2 refer to real and imaginary parts of 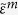 and 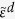, that are the permittivities of metal and dielectric, as 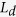 and 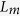 are their thicknesses, respectively; whereas 
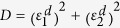
 and 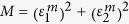. Relations 2 and 3 reduce to the well known expressions 4 and 5 only in the case of low losses (i.e. the terms including 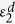 and 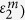:


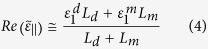



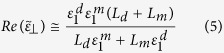


In this case, the simultaneous 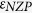 condition occurs when the numerator in Eq. 4 and denominator in Eq. 5 result contemporarily zero, this implying that 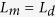 and 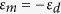 (see Supplementary). These conditions can be fulfilled by selecting materials whose dielectric permittivity values are equal but opposite in sign for a given frequency. Apparently, this condition is verified for many metal-dielectric pairs, nevertheless when losses are included in the analysis, to satisfy this condition becomes challenging. Due to its high losses, gold has to be excluded and, in order to move the desired condition within the visible range, a high index dielectric has to be chosen, thus excluding the most commons 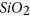 and 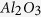. Also 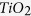 cannot represent a valid alternative, even though this material has been theoretically proposed as a good candidate due to its high index^20^. This because the widely used Palik optical constants for 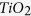 in its birefringent form, greatly decrease after real deposition processes (thermal, sputtering, etc.) from amorphous target or pellets (see Supplementary Informations for thermal 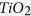 optical constants measured by ellipsometrical analysis). In the present work, silver has been used as metal because of its low loss at visible frequencies, whereas *ITO* (Indium Tin Oxide) constitutes an interesting alternative to a common dielectric, due to its wide utilization in everyday technology, fabrication processes and transparency in most of the visible range and suitably high refractive index. For all these reasons we focused our attention on a *ITO/Ag* pair. As a first step, 20 nm thick layers of *ITO* and *Ag* have been alternatively deposited on a glass substrate up to 5 bilayers, by means of a DC Magnetron technique (see methods). The optical constants of each layer have been separately evaluated by means of spectroscopic ellipsometry and results have been used in the EMT calculations (see Fig. 1b) for a precise simulation of the final metamaterial behavior including optical losses.

## Results and Discussion

**Figure 1:**
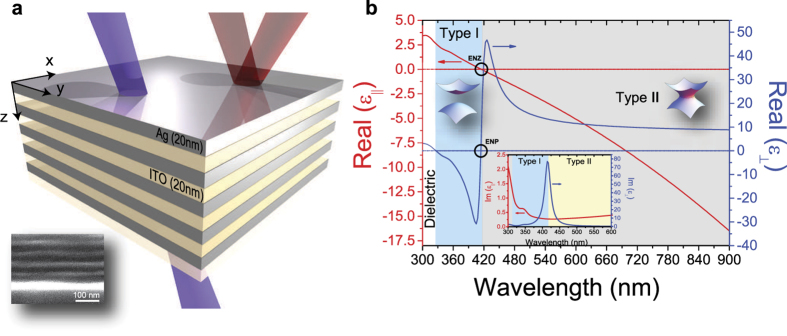
(**a**) Sketch of the obtained HMM structure made of 5 bilayers of Ag (20 nm) and ITO (20 nm). The two laser beams in the sketch represent the different behavior of the obtained material, that is almost trasparent in the UV range, whereas is reflective above 450 nm. Inset is a SEM image of the transversal section of the obtained sample. (**b**) Effective Medium Theory (EMT) of an ITO/Ag multilayer. Real part of epsilon parallel (red curve) and epsilon perpendicular (blue) of the entire structure. In the inset we present the imaginary parts of parallel and perpendicular permittivities. It is evident that the type I/type II transition wavelength occurs at 414 nm.

**Figure 2:**
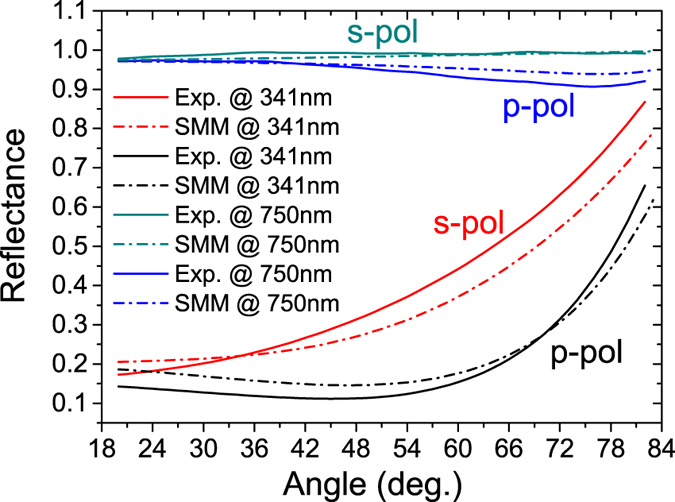
Experimental (solid) and Theoretical (SMM, dash-dot) pseudo-Brewster angle for *s*− and *p*− polarization at two different wavelengths (type I and type II regions). In type II region (at 750 nm), a metallic behavior in 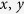 directions causes both the 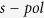 and 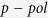 to be completely reflected. In type I region (at 341 nm), the behavior is reversed: a dielectric response with low losses in 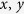 directions allows the appearance of a Preudo-Brewster angle for p-polarized lightwave, as expected.

As shown in Fig. 1b, the medium behaves as a dielectric up to 327 nm because both 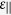 and 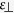 are positive. Between 327 nm and 414 nm, 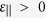 while 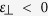, opening a *type I* - HMM window. At *λ* = 414 nm the 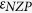 behavior clearly manifests a strong discontinuity in 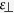, passing from a very high negative value (virtually −∞) to a very high positive one (virtually +∞) while, simultaneously, 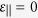. In the inset of Fig. 1b, the imaginary parts of 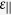 and 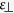 are plotted, showing a very sharp Lorentzian shape for 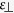, peaked exactly at the transition wavelength. HMM exhibits a *type II* behavior at *λ* > 414 nm. A Scanning Electron Microscopy (SEM) image (inset of Fig. 1a) shows the obtained layer by layer disposition with a fill fraction of almost 50%. Moreover, we have investigated the presence of the “pseudo-Brewster angle”^23^ for two incident wavelengths such as 341 nm (in the middle of the *type I* region) and 750 nm (*type II* region), both for *s−* and *p−* polarization. A clear signature of the *type I* behavior is due to the presence of a pseudo-Brewster angle^20^, while the highly metallic response of the device makes it impossible for the reflectance at any angle to reach values close to zero in the *type II* region. Pseudo-Brewster angle has been theoretically calculated by using a scattering matrix method (SMM) analysis and then the results have been compared with experimental curves obtained by means of variable angle spectroscopic ellipsometry (see Fig. 2).

**Figure 3:**
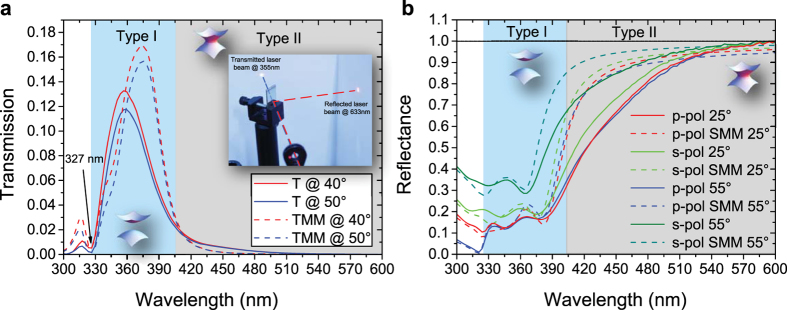
(**a**) Experimental (solid) and transmission simulation (using TMM - dashed) at two different incident angles (40 and 50 degrees). Inset represents the utilization of the obtained device as a HMM-based selective filter. It is well evident the high transmissive behavior in type I region (355 *nm*) with respect the high reflectivity in type II region (633 *nm*). The zero transmission dip, occurring exactly at 327 nm, does not shift when changing the impinging angle, configuring this as a *Ferrel-Berreman mode*. As reported in^24^ this dip is a characteristic of the *epsilon-near-zero* medium, for this it can be used as a signature of the *dielectric/type I* transition. (**b**) Reflectance curves for *s−* and *p*− polarization at angles 25 and 55 degrees (solid lines). Dashed curves have been calculated by means of the SMM model. The zero reflection dip occurring for a p-polarized lightwave at 55°, is a clear evidence of the 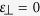 condition^21^, confirming the dielectric/type I transition.

Numerical simulation results (dash-dot curves) are found to be in a very good agreement with experimental results (solid curves). For 341 nm, a pseudo-Brewster angle is clearly visible around 46 degrees for *p–*polarization, while the pseudo-Brewster angle is neither detected nor predicted for *s–*polarization. For 750 nm, reflectance remains extremely high (above 90%) at any angle, for both the polarizations, confirming the high metallic response of the multilayer structure in the *xy* plane. As predicted by Shekhar *et al*.^20^, *type I* - HMMs are more transmissive than *type II*. Indeed, a high transmission window is expected in *type I* region, while high reflectance in *type II*. Theoretical transmission and reflectance behaviors have been calculated by means of a classic TMM and SMM analysis. In order to verify the expected behavior, we performed transmission and reflection measurements by using a V-VASE (J. A. Woollam) spectroscopic ellipsometer. Acquired transmission and reflectance spectra (both experiment and simulation) are shown in Fig. 3a,b, respectively. Transmission has been acquired at two different angles, just below and above the observed pseudo-Brewster angle. For both angles (40 and 50 degrees), a highly transmissive window is clearly visible throughout *type I* region, as predicted by theory. Reflectance has been measured ellipsometrically at two different angles, far below (25°) and close to (55°) the pseudo-Brewster angle, for both *p−* and *s−* polarizations, confirming the low reflectivity of *type I* region in contrast with the high reflectance measured in *type II*. It is worth noting the accordance between simulations and experiments. In the inset of Fig. 3a we report an experiment for the potential application of the metamaterial as a HMM-based selective filter. This application crucially improved our confocal setup while conducting the supercollimation experiment (See supplementary).

Following the experiments proposed by Hoffman *et al*.^21^, we investigated the *dielectric/type I* transition by analyzing the reflection spectrum of both s- and p-polarized plane waves (Fig. 3b). As expected, for an s-polarized impinging light wave, no dip is detected in reflection, while, for a p-polarized light wave, impinging at an angle close to the Brewster one, a dip close to zero is present at this wavelength. When moving away from the Brewster angle, this dip red shifts and, at the same time, moves far from zero, confirming the behaviour described by Hoffman *et al*.^21^ On the other hand, it is possible to detect the *dielectric/type I transition* even more accurately, by investigating the transmission spectrum as well (Fig. 3a). It is worth noting that the narrow dip present in transmission exactly at 327 nm has to be attributed to the so-called *Ferrel-Berreman mode*^24^. Indeed, being a radiative mode, this dip does not shift in wavelength when changing the investigation angle. Furthermore, in ref. 24 it is clarified how this mode appears in correspondence to an *epsilon near zero* medium. The realized system assumes this property exactly at the *dielectric/type I* transition wavelenght, at which 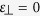. This behaviour is also confirmed by the *Pseudo-Epsilon* measurement (see 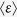 in Supplementary), where the 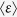 crosses zero exactly at this wavelength. This implies that the occurrence of the *Ferrel-Berreman mode* represents a clear signature of the *dielectric/type I* transition.

Moreover, spectroscopic ellipsometry has been used as a powerful tool to get direct information about the optical constants of the realized structure (see Supplementary). By performing a spectroscopic scan, ellipsometric parameters such as 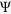 and Δ have been measured and fitted by using an appropriate optical model, to retrieve the values for *n* and *k* for each Ag and ITO thin films. Then it has been possible to calculate the so called *pseudo epsilon*, 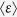. This quantity, whose expression and behavior is reported in supplementary materials, gives us univocal information about the overall optical constants of the HMM.

**Figure 4:**
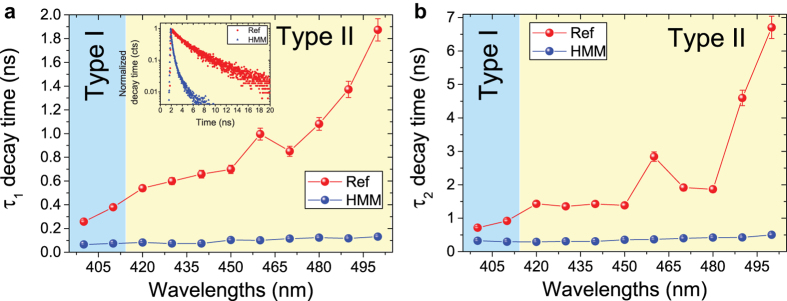
(**a**) Spontaneous emission lifetime 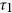 of the C460 dye as a function of emission wavelengths on reference (red circles) and HMM (blue circles) samples. Inset shows the decay time curves (in log. scale) for ref. and HMM, evidencing the spontaneous emission rate enhancement of dye on HMM with respect to ref. sample. (**b**) 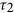 lifetime of C460 dye on reference (red circles) and HMM (blue circles) samples. Solid line is eye guide.

A further investigation has been performed to reveal the existence of high-k modes in the fabricated HMM^25^,^26^,^27^. Fluorescence lifetime measurements, as a function of emission wavelength, have been carried out by means of an ultra-fast time correlated single photon counting (TCSPC) set-up (see Methods). Short-living excitonic states of the chromophores placed in close proximity to the multilayers and measured in the *type I* ad *type II* regions, would represent a clear signature of the presence of high-k modes. The studied HMM structure consists of a Coumarine 460 (C460) dye (0.3% by wt. in ethanol solution) dissolved polymer (PMMA) layer 100 nm thick on top of the 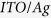 multilayer, (see Supplementary Fig. S3). The maximum emission wavelength of C460 dye dissolved PMMA is observed around 433 nm for an excitation wavelength of 375 nm (see Supplementary Fig. S3c). The fluorescence lifetime measurements of two samples, such as reference sample (C460 on a glass substrate) and HMM (C460 on the HMM) are summarized in Fig. 4 (Supplementary Fig. S3d and S3e for details).

**Figure 5:**
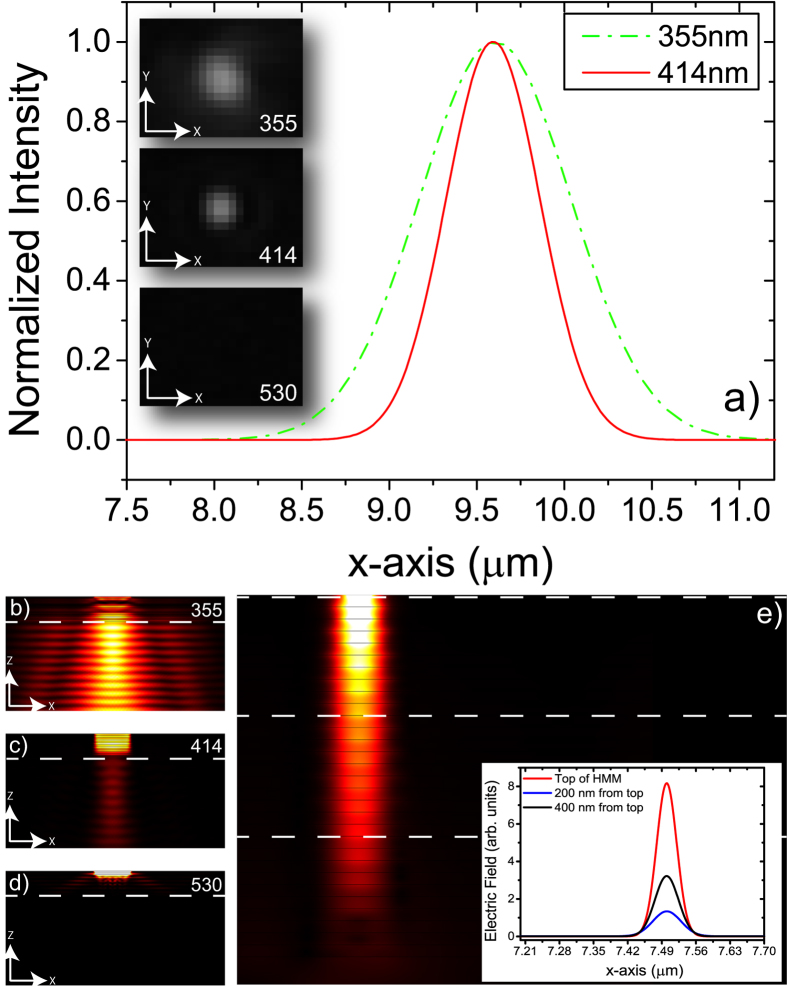
(**a**) Cuts of the transmitted beams acquired in confocal regime at three different incident wavelengths (355 (red solid line), 414 nm (gree dashed line), and 530 nm). Transversal profile intensity at 530 nm results almost zero. Insets are the experimental spots obtained through the 5 ITO/Ag bilayers. (**b**–**d**) FEM simulations of the transmitted field from a 500 nm width slit on the top of a 5 bilayer HMM at the same excitation wavelengths. Maximum of transmission is reached at 355 nm, but no collimation is visible (**b**). For 414 nm, transmission decreases, but a supercollimation effect takes place (**c**). At 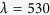 nm no field intensity is detected, but the typical cone profile is appreciable (**d**). We measured a reduction of about 40% in FWHM in the case of 414 nm as compared to 355 (from 1 micron to 0,610 microns). (**e**) represents the FEM analysis of the confinement of light coming from a 40 nm slit placed at the top of the structure, that propagaes as a waveguide for more than 100 Rayleigh lengths.

The data have been fitted using three exponential functions (see Supplementary), through which we identified a longer time (*τ*_3_) attributed to dye molecules away from the HMM, whereas shorter decay times (*τ*_1_ and *τ*_2_), being related to strong coupling of molecules with HMM structure, were used to predict the decay rate enhancement in HMM with respect to ref. sample^28^. Note that same behavior is observed for both lifetimes (Fig. 4a for *τ*_1_ and Fig. 4b for *τ*_2_). Dye on reference sample shows an increase in lifetimes with emission wavelength. However lifetime of C460 onto HMM is almost constant in both the *type I* and *type II* regions of the emission spectra. The observed shortest lifetimes in the HMM based sample are due to the coupling to the high-k metamaterial states present in the metastructure^25^. These results definitely show a spontaneous emission rate enhancement in both regions, as expected in both type I and type II regions. Within the hyperbolic frequency range, propagation of waves is still allowed, but this happens along a preferred direction tilted at some angle, better known as *resonance cone* (RC) angle^29^,^30^,^31^,^32^. Half of the RC angle can be calculated as 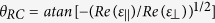. From this equation, it is clear that if 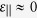, or 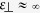, then 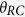 is equal to zero and the wave propagates as a single straight beam, highly confined inside the structure (see supplementary information). This condition is known as *canalization regime*^29^,^33^, and can bring to a supercollimation effect. In order to verify the supercollimation properties of the presented HMM configuration, we performed confocal microscopy experiments. Acquired images at three different incident wavelengths, together with simulations, are reported in Fig. 5.

**Figure 6:**
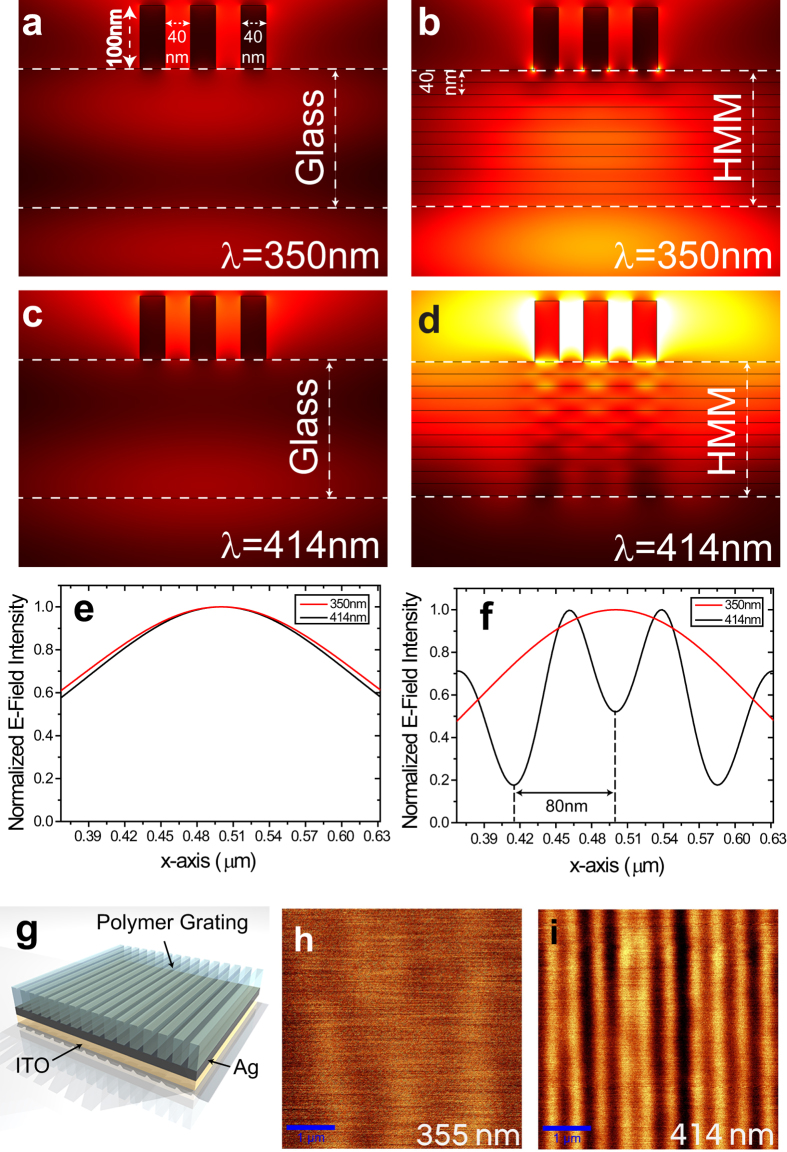
(**a**,**c**) represent the FEM simulations of a plane wave propagating from a slit of 500 nm at the top of a three PMMA nanometric elements (100 nm width, 10 nm separation distance) through a glass slab of thickness 280 nm, at two wavelengths (355 and 414 nm). (**b**,**d**) represent the FEM simulations with the glass slab replaced by the HMM made of 5 bilayers (Ag-ITO 20–20 nm). As appreciable from the transversal intensity profiles made at the exit of the glass/HMM (**e**,**f**), the system is able to resolve the nanometric structure with a resolution comparable to the step of the metamaterial^33^. (**g**) represents a sketch of an experiment made on a single Ag/ITO bilayer with a grating on the top (250 nm stripes and 250 nm gaps). (**h**,**i**) represent the confocal images of the periodic structure at *λ* = 355 nm (**h**) and 414 nm (**i**).

The transmitted light spots reported in the insets of Fig. 5a have been acquired by means of a confocal analysis (see Methods). The red (solid) and the green (dashed) curves represent the normalized transversal intensity profiles of the incident pulsed beams at 414 and 355 nm, respectively. Transversal profile of the confocal image at 530 nm is not reported because transmitted intensity resulted almost zero at this wavelength. It is well evident that the full width at half maximum (FWHM) of the spot at 414 nm is much narrower than that one at 355 nm, thus confirming a supercollimation behavior. Figure 5b–d represent the 2D FEM simulation related to the propagation of a plane wave at three different wavelengths, corresponding to type I (355 nm), canalization wavelength (414 nm) and type II region (530 nm). Light coming from a 500 nm slit placed at top of the first layer propagates through the realized HMM. It is clear that a maximum of transmission is reached at 355 nm, but no collimation is visible. For 414 nm transmission decreases, but a supercollimation effect takes place, corresponding to a “solitary wave” - having the size of the slit - propagating through the HMM structure. At 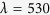 nm no field intensity is detected neither in the experiments nor in the simulations, but the typical resonance cone intensity profile is clearly appreciable, providing a strong signature of the type II metamaterial behavior. The ability of the proposed HMM to confine the light with a resolution given by the step of the structure^33^ is also investigated by means of FEM simulations. As shown in Fig. 5e, the proposed metamaterial is able to confine the light coming from a 40 nm slit placed at the top of the structure for more than 100 Rayleigh lengths. As a result, this device is the perfect candidate for plasmonic, deep-subwavelength bulk waveguides. As previously mentioned, in the “canalization regime” the same HMM structure can be used as a perfect lens. A subwalength sized object placed in close contact with this device can be easily resolved at its bottom with almost no changing in phase for all the Fourier components, because for 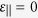 and *ε*_⊥_ = ∞, 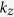 results always equal to zero^34^. Even though Alú *et al*.^35^ and Kyoung *et al*.^36^ reported the possibility of using an 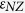 medium as a far field perfect lens, in 2003 Ramakrishna *et al*. have demonstrated the possibility of using an extremely anisotropic medium to achieve the 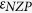 condition, under which the medium shows 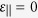 and *ε*_⊥_ = ∞ simultaneously, proposing such a structure as a near-field perfect lens^34^. In our work we point out a quite different mechanism, by exploiting the unconventional refraction conical pattern that light undergoes inside the HMM and by forcing the resonance cone angle to be zero, thus allowing to obtain an extreme resolution limited only by the spatial period of the multilayer. As shown in Fig. 1b, in such a regime losses along the extraordinary optical axis (*z–*axis) constitute the main obstacle toward a real device. The possibility of exploiting this structure as a perfect lens is demonstrated by the FEM simulation showed in Fig. 6.

In the presented simulation, three PMMA 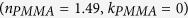 nanometric rectangular elements 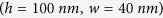, separated by 40 nm, have been positioned on top of the HMM and illuminated by a plane wave at two different excitation wavelengths, 355 and 414 nm. Figure 6a,c represent the 2D FEM simulations in which the HMM has been replaced with a glass layer of the same thickness. In Fig. 6e the transversal intensity profile at the exit of the glass slab for the two wavelengths is reported. It is well evident that information about the nanometric elements is completely lost for both wavelengths. According to our study, by replacing the glass with the 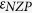 HMM, the behavior reveals a dramatic change (see 2D map in Fig. 6b,d). At the excitation wavelength of 355 nm (Fig. 6f, black dashed curve) the propagation through the HMM structure does not bring any information of the three objects separated by subwavelength distances, as expected for type I - HMM. By exciting exactly at the “canalization wavelength” (414 nm, Fig. 6f, red solid curve), the image of the three nanometric objects is transferred to the exit of the HMM. Being the separation distance between two adjacent objects of only 10 nm, we can ensure that it is possible to distinguish two objects with a resolution down to 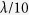. In Fig. 6g we propose an experimental proof of concept of a real system. In order to keep the transmissivity of the device suitably high, a single 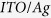 bilayer with a fill fraction of 50% has been realized. We want to stress out that it is well known that even a single bilayer can work as a hyperbolic medium^26^. Figure 6g shows a sketch of the proposed experiment. On the top of the bilayer a simple grating consisting of 250 nm stripes separated by 250 nm gaps has been written by a sophisticated two photon absorption lithography method (see Methods). This grating has been used as the subwavelength image, on the top of the HMM, to be resolved at its bottom. By means of a confocal experiment we tried to resolve the subwavelength grating by illuminating it with the same wavelengths used in the simulation. Results showed in Fig. 6h clearly demonstrate how the information about the subwavelength structure is completely lost for 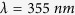 (even though this wavelength is shorter than the canalization one) while the grating is completely visible at the canalization wavelength 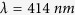, demostrating the possibility of using such a medium as a near field superlens. In conclusion, we demonstrated the design and fabrication of a hyperbolic metamaterial in which the coexistence of two extreme and opposite dielectric anisotropies is verified. The presence of the so-called *Ferrel-Berreman mode* has been both theoretically and experimentally verified, confirming either the *epsilon-near-zero* regime and the *dielectric/type I* transition. The simultaneous presence of a zero and a pole (respectively in the in-plane and out-of-plane dielectric permittivities 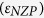 HMM) at 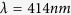, has been theoretically predicted and experimentally observed. At this frequency the obtained dielectric singularity provides suitable conditions to experimentally achieve the so-called *canalization regime*, thus enabling the super-collimation effect precisely at this wavelength. Moreover, here we show how were able to exploit the properties coming from this regime to realize many fascinating devices, such as a HMM-based band-pass filter, manifesting performances far better than the commercial ones. We explain that, within this regime, it is possible to reach previously unpredictable light confinement in the bulk of a hyperbolic metamaterial, forcing a light beam with suitable frequency to propagate through the structure as a solitary wave. Therefore, a bulk nano-waveguide, on micron depths has been properly designed at this particular wavelength. The same device can be exploited as a near field “perfect lens”, capable to reach a super-resolution down to 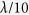, limited only by the step of the metamaterial structure.

## Methods

As a first step we engineered the metamaterial by means of the widely accepted EMT analysis^37^. It is worth noting that the optical constants used in this step have been extracted ellipsometrically directly from the original deposited layers.

### Sample fabrication and measurements

The final multilayer structure has been realized by means of a Physical Vapour Deposition (PVD) technique, by alternating 5 bilayers, each of which made of a transparent oxide and a metal. In particular a 20 nm thick layer of Indium Tin Oxide (ITO) has been deposited on a glass substrate by means of a DC Magnetron sputtering system (Edwards Auto 306), with a power of 40 W for 2 minutes at a distance of 7.2 cm between the substrate and the ITO target. Then, an Ag layer (20 nm thick) has been deposited on the ITO using 10 W of power for 3 minutes. This constitutes the bilayer. For each deposition session a control sample has been placed side by side with the main one, allowing to check the optical properties of each sputtered layer, as well as its thickness and uniformity, at the end of each deposition. Spectroscopic ellipsometry has been used to perform this analysis by means of a V-VASE Ellipsometer (Woollam Co.). By setting up a multistep deposition procedure we were able to realize the complete multilayered metamaterial (see Fig. 1a, inset). Its good morphology has been investigated by means of the scanning electron microscopy analysis (SEM). Indeed, ellipsometry has been used as a powerful tool to perform angular transmission and reflection characterizations, as well as the Brewster angle evaluation. The 1D polymer grating on the top of the HMM structure, used to prove the perfect lens behavior of the obtained device, was obtained by a direct writing process based on a laser lithography system (by Nanoscribe GmbH) that enables true 3D micro- and nanofabrication via two-photon polymerization. The instrument (a Photonic Professional GT) combines two writing modes in one device: an ultra-precise piezo mode for arbitrary 3D trajectories and the high-speed galvo mode for fastest structuring in a layer-by-layer fashion.

### TCSPC Measurements

An ultrafast optical set up has been used to measure the lifetimes of the samples. The optical set up consists of a Ti: Sapphire tunable femtosecond laser (Chameleon Ultra II, by Coherent Inc.), Pulse Picker (by Coherent), Second Harmonic Generator (by Coherent), and a spectrofluorometer for time-correlated single photon counting (TCSPC) instrument (by Edinburgh instruments). The time resolution of the TCSPC instrument is ≤5 ps. In the experiments, the Coumarin 460 dye was excited at 370 nm wavelength by using a pulsed laser with a pulse width of about 120 fs and a repetition rate of 4 MHz. Here the emission wavelengths were varied using a monochromator that belongs to the TCSPC instrument.

### Confocal Analysis

A confocal analysis allowed us to verify the supercollimation properties of the HMM. The experiment has been carried out on a WITec alpha 300S Scanning Near Optical Microscope (SNOM), in confocal configuration. For both the excitation wavelengths (355 nm and 414 nm) a Ti:Sapphire tunable femtosecond external pulsed laser (Chameleon Ultra II, by COHERENT Inc.) has been duplicated in frequency (by second harmonic generation) and focused on the sample through a Zeiss 100x objective (0.75 of numerical aperture (NA)) confocal with a Leica 50x objective (0.5 NA), used at the bottom for collection. We ensured that both the energy and polarization of the external beam was the same for the two excitation wavelengths. Final detection was performed by means of a CCD camera.

### Modeling

In order to perform transmission and reflection simulations, we set up a classic Transfer and Scanning matrix method (TMM and SMM) code, implemented in MATLAB. Our code automatically takes into account the contribution of air, as an incoming medium and glass as an outgoing medium. Supercollimation and Perfect Lens simulation were performed by means of a Finite Element Method based code.

## Additional Information

**How to cite this article**: Caligiuri, V. *et al*. Dielectric singularity in hyperbolic metamaterials: the inversion point of coexisting anisotropies. *Sci. Rep.* **6**, 20002; doi: 10.1038/srep20002 (2016).

## Supplementary Material

Supplementary Information
