# Supplementary material for: Dielectric singularity in hyperbolic metamaterials: the inversion point of coexisting anisotropies
Source: Sci Rep. 2016 Feb 2;6:20002. doi: 10.1038/srep20002 (PMC4735793; doi:10.1038/srep20002)
Supplement: Supplementary Information [file srep20002-s1.pdf]

Supplementary Information - Dielectric singularity in Hyperbolic Metamaterials: the inversion point of coexisting anisotropies

V. Caligiuri[1], R. Dhama[1], K. V. Sreekanth[2], G. Strangi[2,1,*] & A. De Luca[1,*]

[1] *Department of Physics and CNR - NANOTEC, University of Calabria, 87036 Rende Italy*

[2] *Department of Physics, Case Western Reserve University, 44106-7079 Cleveland, USA*

**e-mail: antonio.deluca@fis.unical.it; gxs284@case.edu*

1 Effective Medium Theory

Optical constants of the HMM structure are designed by using the well known Effective Medium Theory (EMT). For a multilayer configuration, the in-plane and out of plane dielectric permittivities of the entire structure are approximated as in eq. 1 and eq. 2:

$$\tilde{\varepsilon}_{\parallel} = \frac{L_d\tilde{\varepsilon}_d + L_m\tilde{\varepsilon}_m}{L_d + L_m}; \tag{1}$$

$$\tilde{\varepsilon}_{\perp} = \frac{\tilde{\varepsilon}_d\tilde{\varepsilon}_m(L_d + L_m)}{L_d\tilde{\varepsilon}_m + L_m\tilde{\varepsilon}_d}; \tag{2}$$

The complex nature of the dielectric permittivities of the two basic materials (m for metal and d for dielectric) is specifically reported in order to underline the fundamental role played by the imaginary parts. Expressions 1 and 2 can be manipulated in order to separate their real and imaginary parts as follows:

$$\tilde{\varepsilon}_{\parallel} = \frac{\varepsilon_1^d L_d + \varepsilon_1^m L_m + i(\varepsilon_2^d L_d + \varepsilon_2^m L_m)}{L_d + L_m} \tag{3}$$

$$\tilde{\varepsilon}_{\perp} = \frac{L_d + L_m}{(\varepsilon_1^d L_m + \varepsilon_1^m L_d)^2 + (\varepsilon_2^d L_m + \varepsilon_2^m L_d)^2} \left\{ \varepsilon_1^m L_m D + \varepsilon_1^d L_d M + i \left[\varepsilon_2^m L_m D + \varepsilon_2^d L_d M \right] \right\} \tag{4}$$

Here subscript 1 and 2 are referred to real and imaginary parts, L_d and L_m are the thicknesses of dielectric and metal, respectively, whereas $D = (\varepsilon_1^d)^2 + (\varepsilon_2^d)^2$ and $M = (\varepsilon_1^m)^2 + (\varepsilon_2^m)^2$. In the case of negligible losses these two equations can be reported to the easier expressions:

$$\varepsilon_{\parallel} = \frac{L_d \varepsilon_d + L_m \varepsilon_m}{L_d + L_m}; \tag{5}$$

$$\varepsilon_{\perp} = \frac{\varepsilon_d \varepsilon_m (L_d + L_m)}{L_d \varepsilon_m + L_m \varepsilon_d}; \tag{6}$$

It is worth noting that this approximation is not always allowed. A characteristic example in which these relations can not be applied is the case of Ti based HMMs. From a careful analysis of eq. 5 and 6, it results that a simultaneous singularity in $\varepsilon_{\parallel}$ and $\varepsilon_{\perp}$ can be found by solving the following system:

$$\begin{cases} L_d \varepsilon_d + L_m \varepsilon_m = 0; \\ L_d \varepsilon_m + L_m \varepsilon_d = 0; \end{cases} \tag{7}$$

Solutions for this system is only possible when simultaneously $L_d = L_m$ and $\varepsilon_d = -\varepsilon_m$, a condition that we called as “Epsilon Near Zero and Pole” (ε_{NZP}). It is clear that the highest the refractive index of the dielectric, the more red shifted the (ε_{NZP}) point will be. Due to its high refractive index in its birefringent form, TiO_2 seems to be a good candidate for pushing the (ε_{NZP}) point far within the visible range. Unfortunately, when depositing this material by means of classic physical deposition techniques (sputtering, thermal, e-beam evaporation, etc.) from amorphous targets or pellets, TiO_2 loses its birefringence, turning into an amorphous low index dielectric. In figure ...

a comparison between the well known Palik's optical constants inherent to the birefringent rutile and a thermal evaporated 20nm thick amorphous TiO_2 we deposited is given, in order to show the dramatic lowering of the dielectric permittivity that, in fact, pushes the transition wavelength in the near UV.

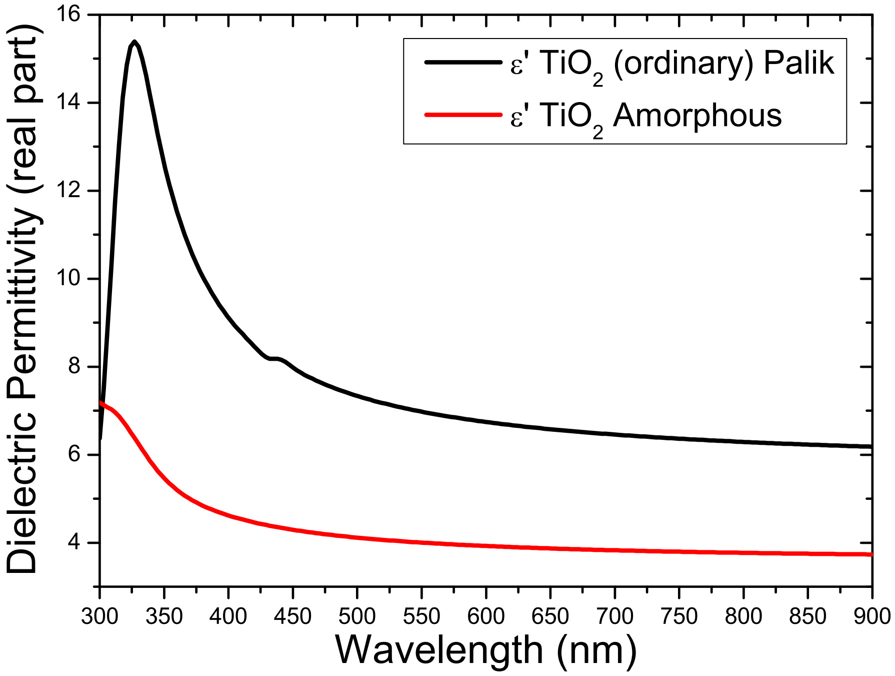

Figure 1: Real part of Palik's well known dielectric permittivity for TiO_2 in its birefringent form (ordinary axis) compared to that one measured on a thermally evaporated thin film from amorphous pellet. The significant lowering showed in the picture dramatically blue shifts the transition wavelength in the UV range.

2 Ellipsometry

Optical constants of realized thin films have been characterized by using spectroscopic ellipsometry. This analysis consists in the evaluation of the ratio $\rho = r_p/r_s$ between the p-polarized and the s-polarized waves reflected from a thin film, deposited on a known substrate. Starting from this measurement, it is possible to evaluate the ellipsometric angles Ψ and Δ by simply applying the well known relation $\rho = tan(\Psi)exp(i\Delta)$. The construction of an optical model for each deposited layer, and the consequent fit of the experimental data for Ψ and Δ, permits to extract the precise optical constants of the analysed layers.

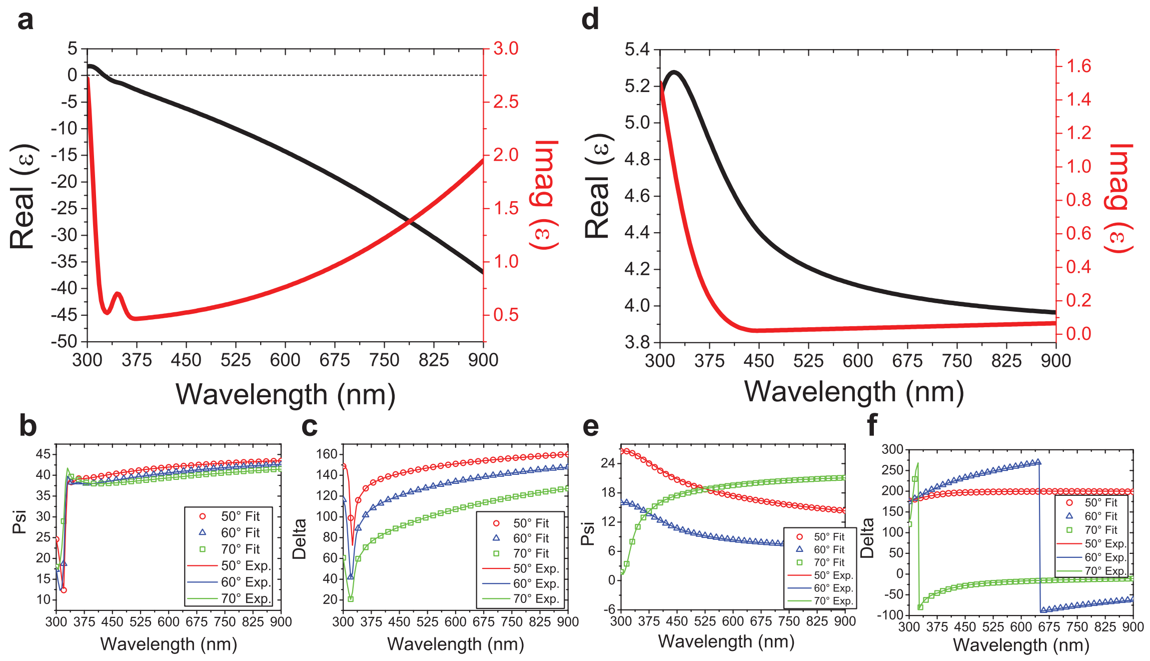

Figure 2: Real and Imaginay parts of optical costants for Ag and ITO. **a** and **d** represent the optical constants of Ag and ITO layers, respectively, found after a fit of the ellipsometrical angles Ψ and Δ reported in **b** and **c** for Ag, and **e** and **f** for ITO, respectively. The ellipsometrical angles Ψ and Δ measured (solid lines) and fitted (scattered data) for Ag and ITO layers are reported for three different incident angles, 50 (cirlces), 60 (triangles) and 70 (squares) degrees, respectively.

The optical constants obtained for each Ag and ITO layer of the HMM are shown in Fig. S2a and S2d, where the values of Ψ and Δ used to find them are reported in Fig. S2(b-c) and S2(e-f) for Ag and ITO, respectively. The best fit for thicknesses extracted from the optical model, as well as the optical constants, are used to perform an accurate EMT design of the entire HMM device. The ellipsometrical angles Ψ and Δ have been measured and fitted also for the final Ag/ITO multilayered HMM. Results are plotted in Fig. S3a.

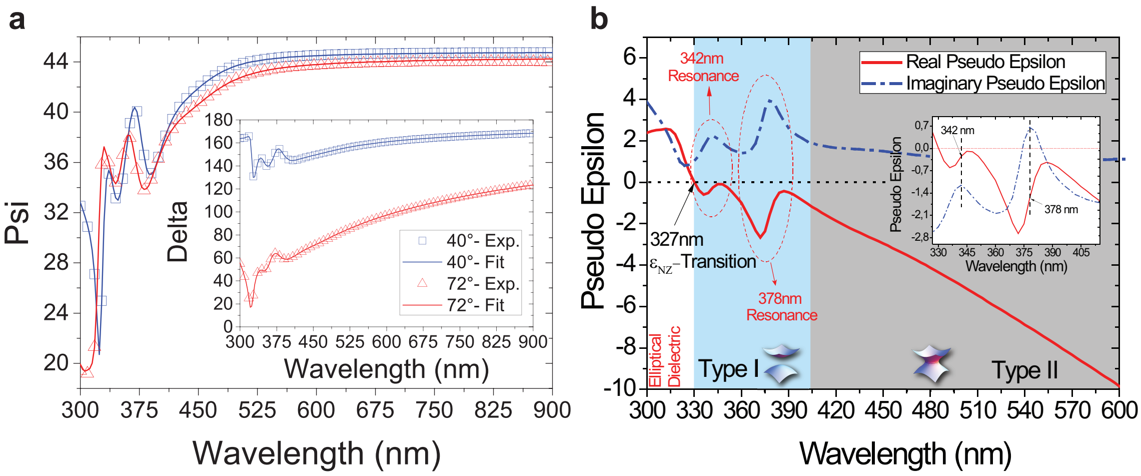

Figure 3: **a** Ellipsometrical constants Ψ and Δ measured (scattered data) and fitted (solid lines) of the whole hyperbolic metamaterial. It is worth noting the good accordance between measures and fitted data. **b** Pseudo Epsilon measured ellipsometrically for the final HMM.

This procedure gives us precious information upon the optical behaviour of the hyperbolic metamaterial. In particular the so called "*Pseudo Epsilon*" can be extracted. This quantity, strictly connected to $\varepsilon_{||}$ and $\varepsilon_{\perp}$, is expressed as:

$$\langle \varepsilon \rangle = sin(\theta)^2 \left[1 + tan(\theta)^2 \left(\frac{1 - tan(\Psi)}{1 + tan(\Psi)} \right)^2 \right] \quad (8)$$

Measured Pseudo Epsilon for the whole multilayer is reported in Fig. S3b. Many useful information can be extracted from this quantity. First of all, it becomes negative exactly at 327nm, confirming the transition from pure dielectric to Type I - HMM. Then, two resonances are clearly visible at $\lambda = 342nm$ and $\lambda = 378nm$, corresponding to two flexes in the real part of $\langle \varepsilon \rangle$, and two lorentzian shaped peaks in the imaginary part. It is worth noting that the maximum of transmission (obtained in the Type I region) occurs exactly in the middle of these two resonances.

3 Time-resolved photoluminescence measurements

To evidence the existence of high-k modes in the fabricated HMM, we performed fluorescence lifetime measurements as a function of emission wavelength. The fabricated HMM structure consists of a Coumarin 460 (C460) dye (0.3% by wt. in ethanol solution) dissolved polymer (PMMA) layer of 100 nm thickness on top of a 5 pairs of ITO/Ag stack (Fig. S4b). A reference sample was used to compare the results, which consists of C460 dye dissolved PMMA layer (100 nm thickness) on a glass substrate (see Fig. S4a). An ultrafast optical set up has been used to measure the lifetimes of the samples. The optical set up consists of a Ti: Sapphire tunable femtosecond laser (Chameleon Ultra II from Coherent), Pulse Picker (by Coherent), Second Harmonic Generator (by Coherent), and a spectrofluorometer for time-correlated single photon counting (TCSPC) instrument (by Edinburgh instruments). The time resolution of the TCSPC instrument is $\leq$ 5 ps. In the experiments, the C460 dye-doped PMMA layer was excited by using a pulsed laser at 375 nm with a pulse width of about 120 fs and a repetition rate of 4 MHz. The fluorescence emission of reference sample is reported in Fig. S4c. The maximum emission is observed at 433nm. The fluorescence time decay curves of reference and HMM samples are shown in Fig. S4d and S4e. It is very clear from the figure that there is a large variation in time decay for HMM compared to reference sample, and these differences are more pronounced at an emission wavelength of 500nm (the type II region). In order to obtain the lifetime, the data are fitted using three exponential functions, $R(t) = B_1 exp(-t/\tau_1) + B_2 exp(-t/\tau_2) + B_3 exp(-t/\tau_3)$, with τ_i being the decay times. Since longer time (τ_3) is attributed to dye molecules away from the HMM, we use shorter decay times (τ_1 and τ_2) to predict the decay rate enhancement, these shorter decay times being related

to strong coupling of molecules with HMM structure.[1] The obtained shorter lifetimes (τ_1 and τ_2) of reference and HMM samples as a function of emission wavelength are shown in Fig. 4 in the main text. The observed response of HMM definitely supports the existence of high k-modes in the fabricated ITO/Ag HMM.

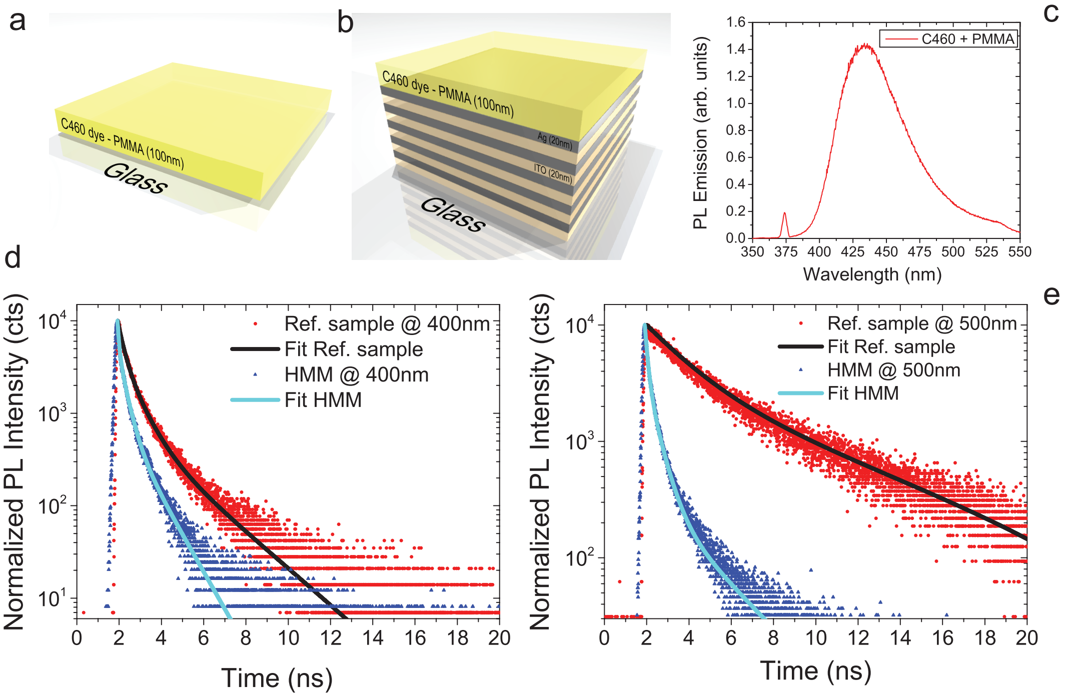

Figure 4: **a** and **b** are the sketches of Ref. and HMM samples, respectively. **c** represents the fluorescence emission of C460 dye-doped PMMA from the reference sample, presenting a maximum around 433nm. **d** and **e** represent decay times at two different emission wavelengths, 400nm (type I region) and 500nm (type II region), respectively.

It is well known that a stringent signature of a topological transition (dielectric/type II, for example) can be deduced by probing the spontaneous emission rate of a fluorophore put in close proximity of the top of the structure. In their seminal work (see ref. [2]), Krishnamoorthy et al.

explain that such a signature can be predicted by calculating the so called topological parameter α, defined as follows:

$$\alpha = \frac{2\sqrt{|\varepsilon_{||}|\,\varepsilon_{\perp}}}{1 + |\varepsilon_{||}|\,\varepsilon_{\perp}} \tag{9}$$

The decay rate for such a HMM is dominated by the contribution from the high-wave vector states, and can be calculated as follows:

$$\Gamma_{high-k} \approx \frac{\mu_{\perp}^{2}\alpha}{8\hbar d^{3}} \tag{10}$$

Where $\mu_{\perp}$ is the dipole moment of a perpendicularly oriented dipole and d is the distance of the dipole from the interface. To demonstrate this transition, here we want to consider the same calculation, based on the effective medium approximation. Figure S5 shows both the topological parameter (red line) and the spontaneous emission rate (black line) calculated for our structure. Moreover, an in depth analysis of the fluorescence lifetime, reported in the main text, is plotted here (see inset of figure S5), showing the sharp reduction in the spontaneous emission lifetime expected around the transition wavelength. The inset represents a zoom of the lifetime τ_1 reported in figure 4a of our main text. The results are in complete agreement with Krishnamoorthys work, thus confirming that the enhancement of the spontaneous decay rate of the top placed fluorophore is as expected at an epsilon-near-zero transition. What we want to underline is that, for the first time, the enhancement of the decay rate in a type I hyperbolic metamaterial is reported, showing experimentally that, due to the hyperbolic nature of the medium, an enhancement of the decay rate can be achieved also in this range.

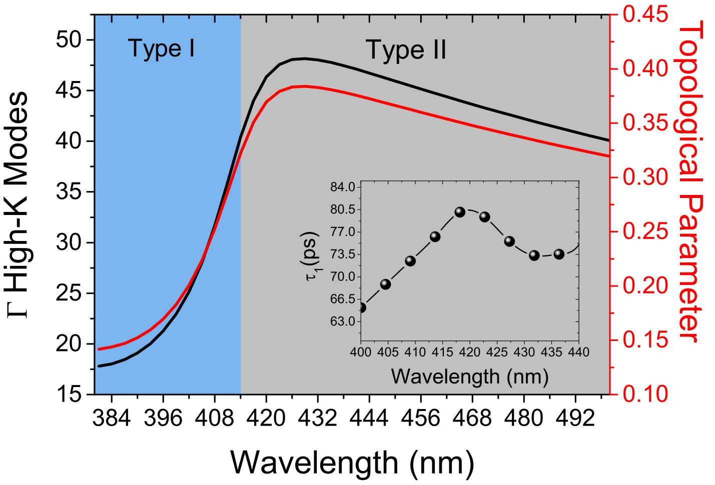

Figure 5: Topological parameter (red line) and fluorescence decay rate (black line) behaviours, calculated for the proposed ε_{NZP} HMM, based on the relations reported in Krishnamoorthy's work. In the inset, a zoomed portion of the measured fluorescence lifetime, already reported in the main text, demonstrates the predicted enhancement of the fluorescence decay rate also in proximity of the ε_{NZP} transition.

4 Field Propagation inside HMMs

The optical properties of a general photonic structure can be described by means of its tensor Green function [3]. In its general form the Green function may present, in the hyperbolic frequency region, singularities responsible of many important properties of such materials, like diverging density of states and Purcell factor. Moreover, in both the two hyperbolic regions, Type I and II, the electric field propagates along a typical cone shaped pattern[4], respecting the condition $\varepsilon_{||}sin^2(\theta)+\varepsilon_{\perp}cos^2(\theta)>0$, where θ is the half angle between the two lobes of the resonance cone (θ_{RC}. The

values θ_{RC} assumes in the proposed ε_{NPZ} HMM for the whole visible range can be calculated straightforwardly from the previous relation, as follows:

$$\theta_{RC} = atan\left(\sqrt{-\frac{Re(\varepsilon_{\parallel})}{Re(\varepsilon_{\perp})}}\right) \tag{11}$$

and are plotted in Fig. S6.

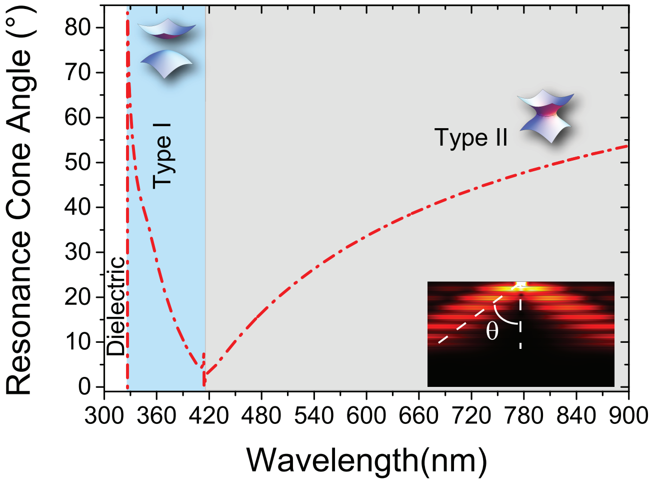

Figure 6: Resonance cones angle theta plotted versus wavelengths. Theta reaches zero exactly in the canalization region occurring at 414 nm. In the inset it is shown the electric field propagation coming from a vertical oriented electric point dipole source at 532nm (Type II region). Electric field inside the HMM splits into two well distinguishable lobes, forming the typical resonance cone pattern exactly at the calculated angle.

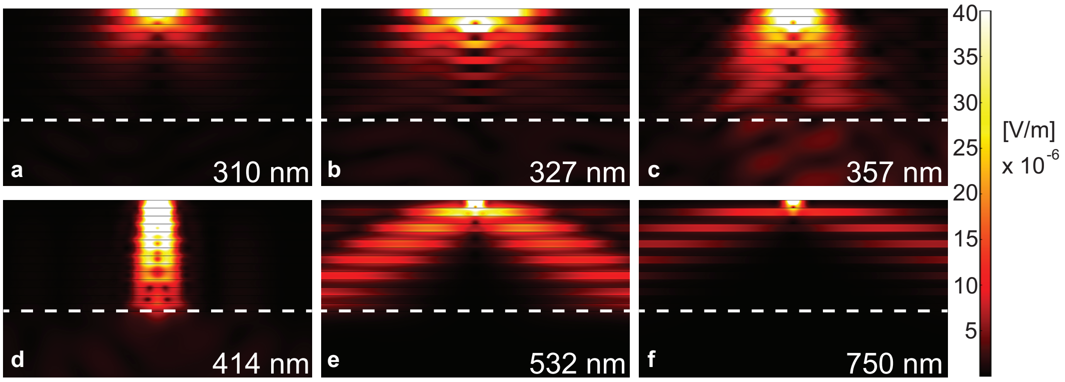

Figure 7: Electric Field propagation inside the HMM for different wavelengths. Simulations have been performed by means of a sophisticated Finite Element Method based code, considering a vertical oriented dipole positioned just at the top of the HMM. Excitations wavelengths lie respectively: **a** In the effective dielectric region. **b** At $\lambda = 327nm$ corresponding to the $\varepsilon_{||}$ near zero condition. **c** In the type I region. **d** In the canalization regime. **e-f** Within the type II region.

In Fig. S7 (a-f) propagation of the electric field emitted by a vertically oriented point dipole inside the proposed ε_{NPZ} HMM is shown for all the characteristic wavelengths. In Fig. S7d the propagation of the electric field inside the HMM at the canalization regime is reported. It is clear that within this region light propagates inside the structure as a straight, deeply subwavelength, well confined "spatial soliton". Such a characteristic allowed us to achieve the so called "Perfect Lens condition". While moving towards type II region, the typical resonance cones appear and, as predicted by eq. 11, the angle becomes wider when moving deeply in the infrared region.

1. Lu, D., Kan, J. J., Fullerton, E. E. & Liu, Z. Enhancing spontaneous emission rates of molecules using nanopatterned multilayer hyperbolic metamaterials. *Nat Nano* **9**, 48–53 (2014).

2. Krishnamoorthy, H. N. S., Jacob, Z., Narimanov, E., Kretzschmar, I. & Menon, V. M. Topological transitions in metamaterials. *Science* **336**, 205–209 (2012).

3. Potemkin, A. S., Poddubny, A. N., Belov, P. A. & Kivshar, Y. S. Green function for hyperbolic media. *Phys. Rev. A* **86**, 023848 (2012).

4. Ishii, S., Kildishev, A. V., Narimanov, E., Shalaev, V. M. & Drachev, V. P. Sub-wavelength interference pattern from volume plasmon polaritons in a hyperbolic medium. *Laser & Photonics Reviews* **7**, 265–271 (2013).
